# A Proposed Methodological Approach for Considering Community Resilience in Technology Development and Disaster Management Pilot Testing

**DOI:** 10.1007/s13753-022-00417-2

**Published:** 2022-06-17

**Authors:** Ioannis Benekos, Evangelos Bekiaris, Katarzyna Wodniak, Waleed Serhan, Łukasz Sułkowski, Hana Gharrad, Ansar Yasar

**Affiliations:** 1 Centre for Research & Technology Hellas (CERTH), Hellenic Institute of Transport (HIT), 15125 Marousi, Greece; 2 Department of Management, University of Social Sciences, Warsaw, 00-842 Warsaw, Poland; 3 Transportation Research Institute (IMOB), Hasselt University, 3590 Diepenbeek, Belgium

**Keywords:** Community resilience, Crisis management, Disaster preparedness and response, Disaster risk management, Search and rescue

## Abstract

Nowadays, resilience has become an indispensable term in several aspects and areas of research and life. Reaching consensus on what actually constitutes “resilience,” “community,” and “community resilience” is still a task that guarantees a vivid exchange of opinions, sometimes escalating into debates, both in the scientific community and among practitioners. Figuring out how to practically apply resilience principles goes even a step further. This study attempts to circumvent the need for a universal agreement on the definition of “community resilience,” which may still be immature, if not impossible, at this time. We accomplish this by proposing a practical methodological approach with concrete methods on how to agree and implement commonly accepted community resilience principles in the context of technology development and pilot testing for disaster management. The proposed approach was developed, tested, and validated in the context of the Horizon 2020 EU-funded project Search and Rescue. Major aspects of the approach, along with considerations for further improvement and adaptation in different contexts, are addressed in the article.

## Introduction and Scope

“Resilience” has always been an integral part of our existence. When “communities” started forming, “community resilience” became an applicable term as well. Even though this topic has been approached by different fields and perspectives through the years, these terms have become increasingly important and used in the scientific community, in light of climate change and related natural or human-made hazards and disasters. This has spurred a proliferation of scientific and technical work by various organizations and institutions operating in this field. This effort is ongoing and dynamically evolving. To cite two characteristic examples:

- The United Nations Office for Disaster Risk Reduction (UNDRR 2017a, p. 3; UNDRR 2017b), formerly the United Nations International Strategy for Disaster Reduction (UNISDR), defines “resilience” as “The ability of a system, community or society exposed to hazards to resist, absorb, accommodate, adapt to, transform and recover from the effects of a hazard in a timely and efficient manner, including through the preservation and restoration of its essential basic structures and functions through risk management.” The UNISDR definition (2009, p. 24) was “The ability of a system, community or society exposed to hazards to resist, absorb, accommodate to and recover from the effects of a hazard in a timely and efficient manner, including through the preservation and restoration of its essential basic structures and functions.”
- The World Road Association (PIARC) defined “resilience” as “A capability to anticipate, prepare for, respond to, and recover from threats with minimum damage to social well-being, the economy and the environment” (PIARC 2015, p. V), whereas proposed amendments to the aforementioned framework that were offered as a substitute included “The ability to prepare and plan for, absorb, recover from, or more successfully adapt to actual or potential adverse events” (PIARC 2019, p. 58).

“Community” is a term that may encompass all sorts of different definitions ranging from geographical locations to various kinds of social networks. The effect of their combination on the resulting term “community resilience” is of increased complexity and vagueness in terms of precise meaning and context. This is also in line with the findings of Ostadtaghizadeh et al. (2015) and Patel et al. (2017), who reported the lack of a common and agreed definition of community resilience.

Our work stemmed from the need to inquire into the gaps that exist in addressing community resilience in the context of disaster management, mainly during the response phase, in the EU H2020 research and innovation project Search and Rescue (S&R).^1^ The aim was to provide high-level directions for the development of technology and wearables used by first/early responders and to guide the operationalization of selected pilots (that is, on-field exercises and simulations), concerning different disaster/emergency situations, where these would be tested so as to enhance community resilience. For this, we developed a participatory and flexible approach, presented herein, that may be adapted and tailored to different contexts.

Thus, it is important to clarify that the scope of our work does not extend to assessing resilience or quantifying it. It rather focuses on the provision of suggestions/recommendations on how identified end user needs and gaps may be best addressed for enhancing “community resilience,” whatever this may mean in a particular context.

The structure for the rest of the article is as follows: Sect. 2 presents the proposed methodological approach and the methods used. It is split in three subsections: Sect. 2.1 presents the approach undertaken in first phase (PHASE A) of our work, which included a detailed literature review of the previously mentioned definitional issues; Sect. 2.2 addresses representative results from the literature search and case study collection and the relevant decisions that guided the subsequent structuring of our approach; and Sect. 2.3 lays out the methodological approach and methods used for the second phase (PHASE B) of work. Section 3 presents key issues reported from the focus group discussion, indicates major findings of the gap analysis (see Chapman 2011, p. 163 for a detailed description of the method) that followed a survey, and describes subsequent refinement and validation insights from a dedicated expert-based workshop where advantages and limitations of our approach were discussed. Section 4 concludes this article by summarizing the major aspects of the work and offering potential future research directions. This article focuses primarily on the proposed methodological approach. Detailed recommendations and suggestions in relation to the concerned technologies/wearables and pilot operationalization will be the focus of a separate article.

## Proposed Methodological Approach and Methods

Based on preliminary literature search as presented in Sect. 1, and the knowledge and understanding from the collective consortium expertise, the methodological approach was split in two distinct phases of work. These phases are next presented and analyzed.

### PHASE A Approach and Methods

PHASE A included a thorough literature review and case study collection that provided the necessary depth to set the scope and context for determining relevant “community resilience” and attributes/capacities. In this context, “attributes” denote quantities of some characteristic or the simple presence or absence of things without any evaluation of quality. “Capacities” may refer to the “evaluation of the performance or quality” (Cutter 2016, p. 748). In the rest of the article, the term “attribute(s)” will be only used to denote either so as to ease the reading when a clear distinction is not needed.

**Fig. 1:**
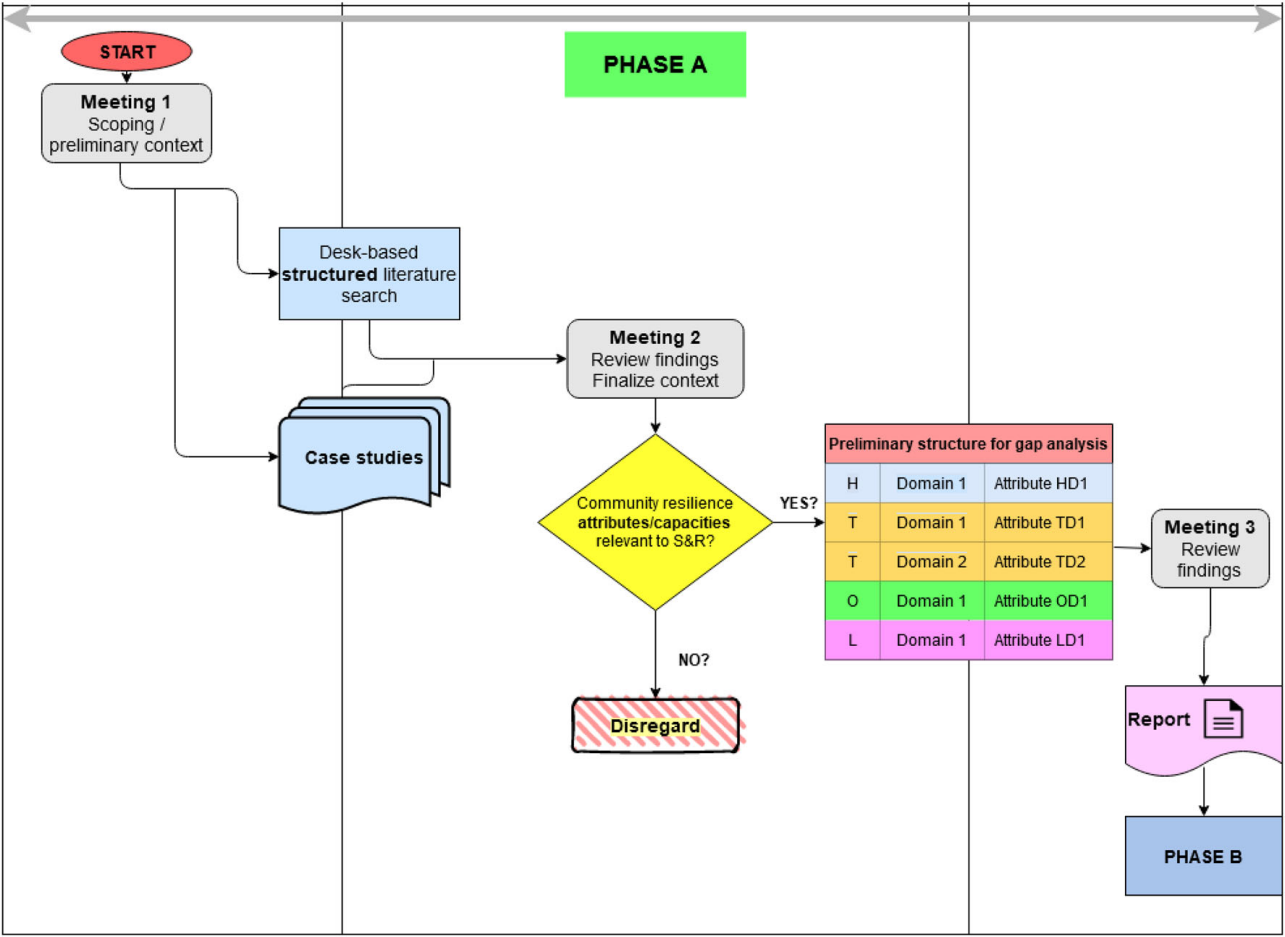
PHASE A flowchart—methodological approach and methods used. *H* human/behavioral, *T* technological, *O* organizational, *L* legal/regulatory, *D* domains/areas within each cluster. Attribute TD2 denotes an attribute that pertains to the Technological (T) cluster and Domain 2 (hence TD2; similar notation for other attributes)

Obtaining and agreeing on a common understanding of what resilience, community, and community resilience mean for the purposes of the S&R project was an important part of PHASE A. Figure 1 presents the indicative sequence, which is summarized in a flowchart. PHASE A is comprised of the following major stages:

1. *Clearly setting scope and context* This stage is very important in order to establish of a common understanding of task objectives and to determine related terminology that is most suitable to the S&R scope of work. Moreover, this effort enhanced efficient and effective communication among the involved parties. Different opinions, perspectives, and (mis)conceptions were presented and discussed.
  *Methods* Stakeholder meetings with expert participation, brainstorming, and a preliminary literature search were used to structure and facilitate the discussions.
2. *Enhancing collective knowledge* This stage finalized the context and deepened the collective knowledge within the pre-established scope of the project. The approach (see Sect. 2.2 for an overview) used at this stage was a combination of two procedures: top-down for the literature search; and bottom-up for the collection of case studies. The top-down approach offered the ability to compare across different scales and units of analysis (for example, local, national, international), whereas the bottom-up approach provided the necessary detail and knowledge/information at a local scale with which to refine the general context (Kruse et al. 2017).
  *Methods* Structured literature review and case study collection based on specific agreed protocols with relevant templates for data collection and processing. Relevant findings were presented and discussed in a roundtable consultation meeting among S&R consortium experts and stakeholders.
3. *Synthesizing and documenting the findings* The literature review and case study collection identified major end user challenges and needs. Absent a commonly agreed definition of community resilience, it may safely be assumed that the way we define it will unavoidably affect any attempt to enhance, let alone to measure, resilience. As such, we emphasized the establishment of a common ground and mutual understanding on how these terms would be most suitable to the S&R scope of work and of practical value to the technology/wearable development (for example, rescue robots and autonomous vehicles, radiation and chemical sensors, first responder prototype uniform for protection and health monitoring capabilities, smart glasses with augmented reality capabilities, and others) and the operationalization of the pilots. We thus proceeded by determining “community resilience” attributes that were found to be relevant and positively contribute to the enhancement of the fundamental “resilience” capabilities of our “community” system in the context of disaster management (Hollnagel 2011; Linkov et al. 2013) as assessed by the experts (see Sect. 2.2.1). It is precisely here that selecting the most appropriate definition for resilience reveals its importance. In our case, due to the strong component of the “community”/social factor, we opted for a definition that emphasizes the adaptive, transformative, and learning aspects of “resilience” (UNDP 2014; UNDRR 2017a; AIDR 2020). The literature provides various configurations for classifying such “resilience” attributes. The configuration matters little; what is important is to include the relevant “resilience” attributes that are most relevant to the studied “community.” In our case, we clustered these by Human/Behavioral (H), Technological (T), Organizational (O), and Legal/Regulatory (L). We further defined some Domains/Areas (D) within each cluster. Figure 1 depicts this clustering in the colored matrix. Subsequently, this matrix was further refined and evolved to a preliminary structure to be used for a gap analysis after presentation of the findings and consultation with stakeholders and consortium partners in a dedicated meeting. The selected attributes are listed in S&R (2020b).
  *Methods* Comparative expert analysis, stakeholder and expert meeting.

These preliminary project components conclude PHASE A and set the ground for identifying the key “community resilience” attributes most applicable to the S&R scope of works.

### Literature Review and Case Study Collection: Major Findings

Next, the major findings from the literature review and the case study collection are presented.

#### Literature Review

The structured literature review relied on expert critical analysis and keywords that considered priority references within the last decade, with a specific focus on the most recent advancements of the last five years. The review aimed at identifying and subsequently finalizing the definitions of “community,” “resilience,” and “community resilience” to be used for the purposes of the S&R project. Additional goals were to identify relevant tools and guidance, and investigate professional-volunteer interactions. After expert screening, a total of 111 sources were considered and analyzed in detail by experts. From these, two thirds (*n* = 66) included scientific and technical papers (33%), Horizon 2020 and FP7^2^ relevant EU-funded projects (12%), reports and/or frameworks (43%) from nongovernmental organizations (NGOs) and national/international organizations (for example, the United Nations, World Health Organization), and key websites of governmental agencies such as the Directorate-General for European Civil Protection and Humanitarian Aid Operations or the U.S. Federal Emergency Management Agency (12%). The remaining third comprised reviews of legislative acts and regulatory rules as well as standardization issues that were included in 45 relevant international agreements, EU Directives, Regulations, and international standards. Preference in source selection was attributed to sources that compiled relevant findings from prior relevant literature search work. It is this group of sources to which we subsequently mainly refer.

Räsänen et al. (2020, p. 6) concluded that “resilience looks notably different when linked to different conceptualizations of community.” They proposed the following categorization in an attempt to distinguish the different notions Räsänen et al. (2020, p. 2):

1. “The community of practice and interest” that comprises networks of specialized professional actors whose role is more prominent in the preparedness and response phases of disaster management;
2. “The interaction-based community” composed of different networks and social groups whose role is more visible in the response and recovery phases; and
3. “The place-based community” that is mainly defined by individuals and social structures within geographical boundaries, whose role may or may not appear in policy documents and is “visible in some of the policy documents” but not always clearly defined or including the same actors and “its role seems to be rather limited” as per Räsänen et al. (2020, p. 5, 6) at least at the countries studied in the aforementioned citation (that is, Finland, Norway, and Iceland).

Often, reports from international organizations and civil protection websites, for example, UNDP (2014), UNDRR (2017a, 2019), FAO (2017), and the Directorate-General for European Civil Protection and Humanitarian Aid Operations (DG-ECHO 2021), refer to communities in the sense of the aforementioned place-based communities, due to their interest in relieving the vulnerable populations that are directly affected by the disaster. As such, they often provide too generic or limited guidance for the development of relevant technologies and their contextual operationalization. Other recent definitions provided by FEMA (2021), Mulligan et al. (2016), DRIVER+^3^ (2017), or that can be inferred from NGOs’ websites, such as Habitat for Humanity,^4^ GDNR,^5^ SARAID,^6^ claim that “community” is a multifaceted concept that evolves dynamically with time, in line with Räsänen et al. (2020). As such, tools and assessments should be adapted to the specific context (IFRC 2016). Therefore, we opted for the community of practice and interest to best apply to the scope of our project. This basically includes first responders (for example, firefighters, emergency medical services, police, coast/border security) and early responders and sustained support elements (for instance, critical infrastructures and infrastructure operators, utility companies, registered national and international voluntary organizations active in disaster response and spontaneous volunteers, public authorities, manufacturers of unmanned aerial vehicles (UAVs), robots/vehicle, and support aid).

In terms of resilience, the scientific literature has long debated about the term and two approaches have mostly prevailed:

1. The engineering approach that is viewed as the more classical approach emanating from physics models and mainly applicable in earlier works in infrastructure engineering systems (H2020-EU 2016a). The concept mainly relies on the bounce-back ability to a predetermined assumed state of stability for the system.
2. The approach that puts more emphasis in assuming that a new state of equilibrium may exist and that a system may shift by adaptation, transformation, and learning to this new state of equilibrium, which is more in line with the notion of ecological resilience (Walker and Salt 2006).

The landscape of resilience definitions and indicators is very wide. Cutter (2016) evaluated 27 disaster resilience assessment approaches. These provided different tools, scorecards, or indices for measuring resilience in different contexts, but offered no consensus on which community attributes to include. Resilience was in various cases conceptualized as a process, a property, an ability, or some combination of these. Studies have warned against the general use of the term when it obscures the importance of the individual elements of which it may be composed (Patel et al. 2017). Recent definitions of resilience or community resilience by the US National Academies of Sciences (NAS 2012), UNDRR (2015, 2017b), DG-ECHO (2021), Public Safety Canada (2017), Wahlström (2015), the World Health Organization (WHO 2017), and the Australian Institute for Disaster Resilience (AIDR 2020) recognize the adaptive, transformative, and learning aspects of resilience, in particular in the context where a strong community component or social aspect is present.

In addition, Mulligan et al. (2016) identified the lack of systematic development of an operationalized model of community resilience. Recent landmark EU-funded projects and/or proposed frameworks were also considered in terms of how these dealt with the issue of resilience and community resilience. Here as well, the approaches differ or may not be specific enough or easily adaptable to the specific context, for example, due to data interoperability and compatibility issues (NASEM 2019), so as to provide specific guidance. In brief:

1. The CoBRA (UNDP 2014) conceptual framework builds on a bottom-up approach on the household level for place-based community resilience building in disasters. It relies on resilience indicators and clearly supports building adaptive capacity through a participatory approach, like we propose. But Community Based Resilience Analysis (CoBRA) neither explicitly defines resilience nor provides guidance for technology development.
2. The emBRACE^7^framework for community resilience (Kruse et al. 2017) builds from empirical evidence obtained from limited and specific geographical contexts (case studies in five countries). It comprises three interrelated domains that shape resilience within the community: resources and capacities, actions, and learning. These domains are embedded in two layers of extra-community processes and structures. It is too generic to provide specific guidance.
3. The European Commission (EC 2018) elaborates on the findings of five landmark EU-funded projects in relation to resilience management of critical infrastructures (CIs): IMPROVER,^8^ RESILENS,^9^ SMR,^10^ RESOLUTE,^11^ and DARWIN.^12^ Here as well, the different notions of resilience used are emphasized and highlighted. IMPROVER puts emphasis on the preservation of key societal functions and RESILENS addresses organizational resilience with a clear view on the bounce-back (absorb and recover) aspect of resilience. SMR considers urban and city resilience and is mainly concerned with the sustainability and continuity of critical services so as to deliver a timely restoration of these. RESOLUTE and DARWIN approach resilience with a view on holism and system complexity, tackling the characteristics of nonlinearity and the emergence of behaviors that cannot be solely understood by analyzing the individual components. RESILENS, IMRPOVER, and RESOLUTE explicitly target the end users of CI services. These projects, although they provide interesting “resilience” management frameworks, are mainly targeted to CIs and not to the “resilience” of the “community” as defined in our context. Consequently, recommendations and guidelines are tailored accordingly.
4. Other recent EU-funded projects with an aim to address societal resilience to disasters are also mentioned in EC (2019). These include i-REACT^13^ and DRIVER+. The i-REACT (H2020-EU 2020) program focuses on multi-risk mapping (preparedness, response, and recovery phases) of cyber technologies rather than resilience. Resilience is not clearly defined. DRIVER+ (DRIVER+ 2017; Merkle et al. 2020) created a portfolio of innovative solutions, available through a dashboard, which focus on coordination during the response phase. DRIVER+ also used a combination of top-down and bottom-up approaches by selecting the Community Engagement Theory (CET) as the theoretical framework within which to inform a “community resilience” measurement method (Paton and Buergelt 2012) as well as the Community Resilience Advancing Toolkit (CART) discussed by Pfefferbaum et al. (2013a, b, 2015) as a practical toolkit to adapt in order to enhance resilience awareness through participatory methods. CET focuses on the psychological/behavioral perspective with very limited consideration on the technological and operational aspects that are crucial in our scope. The CART approach is based on a survey that encourages public engagement in problem solving and suggests the community obtain resilience awareness of the relevant available tools and methods available for building resilience capacity. Our proposed approach focuses on how to best combine community resilience attributes with key user needs (see PHASE B on Sect. 2.3), while adding value to the aforementioned resilience information awareness in the sense that it considers all aspects (human/behavioral; technological; organizational; legal) and is in line with Stage 3 of the CART approach that emphasizes the need to develop goals and objectives in order to provide tailored and practical recommendations.
5. EU-CIRCLE^14^(H2020-EU 2016b) is another EU-funded project that addresses resilience in CIs due to climate change effects. It provides a methodological framework with a number of tools and methods for improving the adaptive capacity of CIs against projected climate change.

Our literature search suggests that it may be of more value and greater practical use to be explicit on the elements of resilience that one is focusing on, based on the specific context of the local system (Cutter 2016; Mulligan et al. 2016; Kruse et al. 2017; Räsänen et al. 2020). This approach was undertaken in our case by weighting these terms (that is, adaptation, transformation, and learning) and the respective resilience attributes for the community of our specific interest (that is, first and early responders), as in Sect. 2.1. Moving from the generic to the specific, we attributed community resilience attributes in relation to fundamental resilience capabilities and features of an adaptive system similar to the approach proposed under the Resilience Analysis Grid (Hollnagel 2011), and the adaptive phases of the Resilience Matrix proposed by NAS (2012) and Linkov et al. (2013). Fusing terms and definitions from these approaches, we specified four clusters of such adaptive system resilience features:

1. *Anticipate/Plan-prepare* Address the potential—lay the foundation to keep services available and assets functioning during a disruptive event;
2. *Monitor/Absorb* Address the critical—maintain the most critical asset functions and service availability while repelling or isolating the disruption;
3. *Respond/Recover* Address the actual and respond to regular or irregular disruptions by adjusting functioning to existing conditions; restore all asset functions and service availability to their pre-event functionality; and
4. *Learn/Adapt* Address the factual by learning from experiences of both successes and failures by using knowledge from the event to become more resilient.

#### Case Studies

The Case Study collection comprised 24 specific cases and an additional 8 general overviews collected from the S&R consortium experts. These consortium partners belonged to the so-called community of practice and interest as defined in Sect. 2.2.1. The cases covered a range of natural and human-made hazards and disasters in Europe and internationally, and included earthquakes, floods, forest fires, fires in urban areas, building collapse, gas poisoning, CBRN (chemical, biological, radiological, and nuclear) explosions, terrorist attacks, and railway accidents, among others.

The findings of the cases studies were derived from a set of questions that revolved around the challenges encountered, lessons learned, and best practices demonstrated in community resilience. The analysis of these case studies resulted in five broad themes related to community resilience in disaster management. Additionally, S&R technologies ran through all these themes. The main aspects of these themes, which are interrelated, are highlighted below.

Under the first theme, technologies, challenges, and gaps identified consisted in a lack of access to communication networks with which to transfer data via Wifi, a lack of reliable maps of an area, saturated phone lines that leave only satellite phones as an option, the limited life of walkie-talkie and GPS batteries, and S&R teams using different radio frequencies, as well as the time-consuming nature of writing down radio communicated messages. Information sharing was also considered a challenge, for example, in relation to evacuation plans, or information that enables determining where it is most important to deploy first responders. Other challenges included a lack of technologies to assist in the safety of the dogs during search operations, and the limited capability of drones and sensors that assess and identify the risks and scope of new explosions, as well as detection of chemical release—all achieved in a quick enough manner. Several solutions were suggested by case study participants to overcome some of these challenges. These suggestions included S&R personnel having compatible and harmonized communication technologies, the unification of communication systems, and the unification of location and data management programs. Other solutions included the improvement of detection tools, such as wearable/portable sensors, radiological meters, and rapid chemical detectors. Some of these solutions seemed possible within existing technologies, such as reserving a separate radio communication frequency for the exchanges between S&R teams.

Second, the case study participants pointed to lack of training as a major challenge for both professionals and volunteers. Participants expressed an opinion that there is a limited number of professionals and volunteers who are adequately trained in emergency response—in particular with respect to professional-volunteer joint-training. They also pointed out that diverse actors are able to complement each other’s skills and expertise through networking. Even if untrained, volunteers can support professionals by performing simpler tasks, such as decongesting the space or evacuating people. One way to tackle the training challenge is by participating in simulations and practical workshops with other emergency teams. For most participants, increased collaboration and training with other organizations provides an opportunity for networking and exposure to different equipment, technologies, knowledge, and practices that create opportunities for knowledge transfer.

Interconnected to training arises the third challenge, coordination. Case study participants indicated their difficulty in activating resources and allocating tasks among multiple actors, taking into account their level of expertise, previous training and experience, as well as their equipment. The participants saw previous training, simulation, and exercises as a major advantage, especially if they were joint actions of all actors involved. This allowed for linking theory and practice while creating joint procedures that allow for more effective coordination. Through this interaction, members of different organizations, both professionals and volunteers, became more familiar with each other, and had better awareness of each other’s’ capacity and expertise, resulting in more effective overall response.

Fourth, linked to coordination is the gap in interorganizational feedback and debriefing. The case study participants pointed out that various actors may be involved in the debriefing separately, rather than in collaboration. The absence of interorganizational debriefing meant that there was no opportunity to analyze the performance from different points of view, which could, in turn, contribute to improved performance and coordination.

Fifth, was the volunteer component of community resilience. Although lack of sufficient training and lack of familiarity with procedures among some volunteers had been identified as a challenge, there was consensus about the participation of volunteers in enhancing community resilience through local knowledge. Volunteers usually have a better knowledge of the disaster area. This applies to the environmental characteristics, for example, the details of a search terrain or the topography of areas affected by fires that do not appear on maps, as well as community characteristics in terms of their needs as well as their skills and expertise. They can help with a correct assessment of the effects of a disaster, the scope of required intervention, and the location of resources. They can explain the work of S&R teams to the affected community, and assist S&R teams in gaining the support of the local community and local authorities. Other advantages of working with volunteers are low personnel cost, strong commitment and motivation, and specialist expertise from the volunteers’ main jobs. Some participants also mentioned that having several actors involved, including professionals, volunteers, and members of the community, can make up for a lack of technology or other resources.

Combined, these five themes offer better insight into what S&R professionals and organizations view to be some of the major challenges or gaps in community resilience, as well as some of the possible solutions. These themes also shed light on what technological developments, innovations, and improvements are needed in disaster management.

The overview of the literature search provided the most characteristic sources based on which we concluded on the use of the terms community, resilience, and community resilience. Due to space limits, the interested reader is referred to S&R (2020b) for the most comprehensive listing of all literature sources, relevant protocols, and templates.

### PHASE B Approach and Methods

PHASE B is built on the context and understanding acquired from PHASE A. That phase identified interlinkages between the community resilience attributes and key end user (community) criteria in an integration exercise that provides targeted and practical recommendations for guiding the design of technologies/wearables and the operationalization of the pilots.

**Fig. 2:**
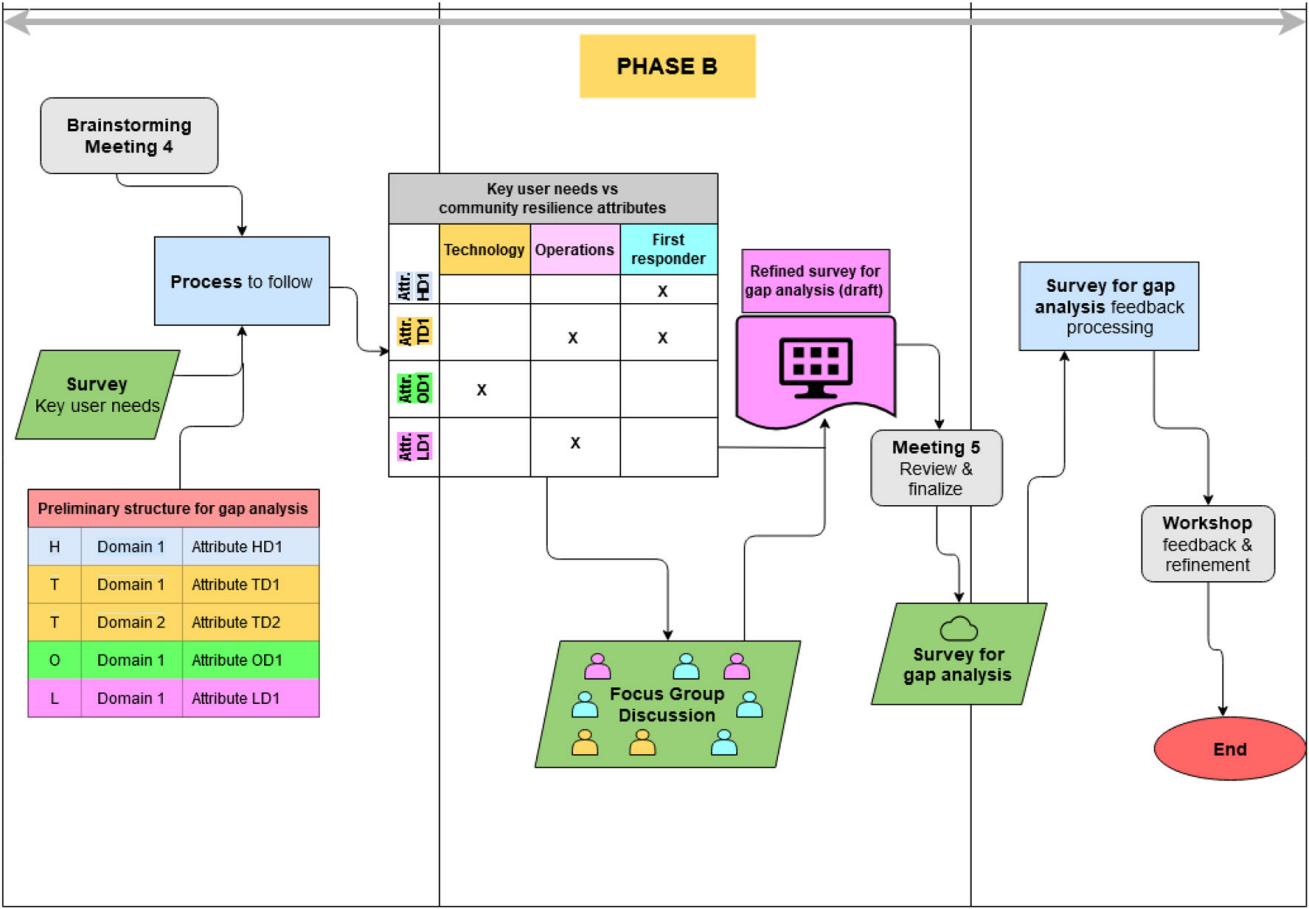
PHASE B flowchart—methodological approach and methods used. *H* human/behavioral, *T* technological, *O* organizational, *L* legal/regulatory, *D* domains/areas within each cluster. Attribute TD2 denotes an attribute that pertains to the Technological (T) cluster and Domain 2 (hence TD2; similar notation for other attributes). *Attr*. Attribute. The different colors in the tables pertain to different clusters

Our methodology relied on a participatory approach that combined several methods. Figure 2 presents the indicative sequence in a flowchart. Major stages are:

1. *Link the community resilience attributes with key user needs* A brainstorming exercise was performed to derive answers from participants in the focus group (FG) discussion, recipients of the survey for the gap analysis, and beneficiaries from the survey responses. Based on previous information, the survey preliminary structure for the gap analysis from PHASE A was further refined so as to obtain information that would be of practical use for the development of the wearables/technologies and the operationalization of the pilots segments of the larger project. In parallel, key community needs were obtained from surveys addressed to end user experts, that is, primarily first and early responders. The interested reader may refer to S&R (2020a) for the detailed survey results. Surveys were limited to consortium partners who were well aware of the specificities of the wearables/technologies to be developed and the pilots to be operationalized in order to obtain targeted and not generic feedback. The following key user needs, grouped into three categories, were selected:
  - First responder education and training (Human/First responder-related)
  - First responder safety (Human/First responder-related)
  - Maneuverability (Operational information-related)
  - Interoperability/Compatibility (Operational information-related)
  - Ergonomy/Ergonomics (Technical information-related)
  - Sensitivity to environmental conditions (Technical information-related)
  - Size of impacted area/victims (Technical information-related)
    Following the identification of key end user needs, a matrix consisting of the community resilience attributes and capacities identified from PHASE A was paired with key user needs (Fig. 2). Participating S&R expert consortium partners then were asked to indicate the relevance they attributed to the different combination pairs of attributes/capacities relative to key user needs. The matrix and the resulting expert feedback are reported in detail in S&R (2021).
2. *Refine the survey structure for the gap analysis and obtain targeted questions*: This task involved three steps:
  - *A pre-FG analysis* In-depth insight was obtained based on the voting of S&R consortium experts for clusters of respondents (first/early responders and others) and in aggregate. From this analysis, the most pertinent community resilience attributes/capacities for user needs in the different categories were identified. This knowledge helped immensely to formulate targeted questions for the FG and to steer the discussion. The pre-FG analysis took into account the detailed background and information obtained from PHASE A as well.
  - *Consortium expert FG* A FG was formed composed of S&R consortium experts with expertise as a first/early responder and/or in technology development, plus an external expert with expertise as a first/early responder. Section 3.1 elaborates on this.
  - *Joint partners and experts meeting* A second meeting with S&R consortium partners and experts reviewed and finalized the proposed updated structure for the GAP survey.
3. *GAP survey and analysis* The survey was administered within the S&R consortium partners. Details concerning survey participation and a brief indicative overview of the major findings from processed feedback are provided in Sect. 3.2.
4. *Refinement of GAP feedback and preliminary validation* A S&R workshop, with several experts, both internal and external to the S&R consortium, was arranged to present the pilots and the relevant technologies/wearables to be developed. A special session was dedicated to the approach followed and the major findings of the GAP survey. In addition to these presentations, the workshop included dedicated roundtable discussions with selected external experts who provided their opinions and suggestions. These observations included key advantages and proposed considerations for future research on the methodological approach. Their positive comments, summarized in Sect. 3.2.2, served as a means for verification and validation of the proposed approach.

## Guiding the Development of Technologies and the Design of Disaster Drills: Results and Discussion on Obtained Feedback

The following sub-sections summarize how the preliminary survey structure for the gap analysis was further refined (Sect. 3.1) so as to produce the final survey that was administered for the gap analysis. The aim was to be as specific as possible in guiding the development of the technologies and the operationalization of the disaster drills where these technologies would be tested. The gap analysis was performed on the received survey feedback (Sect. 3.2). The major findings are briefly presented in Sect. 3.2.1. Strengths and limitations of the proposed approach and the findings were further discussed in a dedicated workshop (Sect. 3.2.2).

### Focus Group Discussion and Finalization of the Survey Structure for the Gap Analysis

Conducting the FG served to refine the preliminary gap structure into the finalized survey for the gap analysis. As explained in Sect. 2.2, the literature review and the collection of case studies allowed us to identify several attributes and key end user criteria for community resilience. These were then combined into a matrix and presented to end users with a request for prioritization. While this activity narrowed down the gap areas to some extent, we still decided to hold a FG that allowed end users to elaborate on the main concepts, summarized into more general questions, with an aim of better understanding the existing gaps in community resilience.

The ensuing FG lasted for two hours and included experts from 13 partner organizations. They addressed 13 questions based on how the 20 end users rated the attributes in relation to the criteria in the preliminary gap structure matrix. The FG results were clustered around five key points of discussion: relations of end users with other organizations; training; health; interoperability; and maneuverability.

The first issue addressed strengthening relations between end users and civil society organizations, local communities, and vulnerable populations. The FG participants emphasized the importance of an active engagement of NGOs and local communities in the entire disaster management cycle, including the early stages of mitigation and preparedness as well as response and recovery. The involvement of these actors should be participatory, according to participants, with decision making delegated to local authorities and representatives of communities. Creation of an endowment plan (budget reserve) for local resources, local volunteers, and NGOs that should be coordinated by a local authority, rather than controlled in a top-down fashion, was indicated as one of the tools for empowering local actors. The participants also pointed to various events, including focus group meetings, gaming scenarios, media communication, and different forms of local outreach, as effective ways to strengthen relations between end users and civil society organizations, local communities, and vulnerable populations.

The significance of training for the improvement of the search and rescue process was the second major issue discussed. The participants highlighted the importance of training in operational procedures as well as technologies, which included being familiar with what technologies are currently available and how to use them in time-constrained situations. An emphasis was also placed on training first responders in disabilities-specific technologies. The participants stressed the significance of incorporating lessons-learned into the training as well.

The third important issue was how to protect the physical and mental health of first responders. When addressing physical health, the participants pointed to wearables providing protection as an obvious response. They also stressed, however, that operational procedures were an important factor in the process. Decision-making and danger assessment procedures were viewed as crucial. Yet many organizations did not seem to have these systems in place, which indicates a gap. Clear decision-making procedures would assist the first responders in their coordination efforts and help to reduce the pressure of having to make decisions on the spot, in time-constrained conditions. Mental health was also addressed in the discussion. Especially emphasized was the cumulative stress on personnel that should be monitored and recognized promptly, another identified gap in this area.

Interoperability in several areas was the next topic. The participants pointed to problems resulting from different organizations using different communication systems, which could be particularly acute when working with volunteers. Non-communicating systems have an impact on locating and allocating resources as some organizations, especially NGOs who work with volunteers, often do not have access to all the necessary information. The participants expressed the opinion that the best way to allocate resources would be through 112^15^ or a Control Centre (CC), which should hold all the necessary information sourced from mobile units via GPS navigation and other call centers. Participants stressed that such CCs should be locally staffed with members of different services such as transport services or civil defense. Sharing terminology and technology, including radio frequencies, was also mentioned in relation to interoperability.

Maneuverability was the last major issue discussed. The participants pointed out that training exercises should be adapted to different equipment. Choosing the right equipment for specific environmental conditions was indicated as crucial. High relative humidity, high temperature, wind, or dust may cause problems with some equipment, such as chemical devices or chemical compounds. Environmental conditions might also cause losing connection with location devices when dogs are in collapsed structures as the available devices are not designed for dogs.

The FG allowed for simultaneous narrowing and deepening of our understanding of the potential gaps in community resilience. The results of the session fed into the final step of designing a gap analysis of the community resilience survey.

### Gap Analysis of the Selected Key User Needs in Building Community Resilience

The survey titled “Gap Survey for Community Resilience in Crisis Management”^16^ was an effort to identify the existing gaps in the key user needs for the developed features (that is, survey questions) that can lead to improvement in the development technologies and procedure operationalization used in crisis management.

The survey was comprised of 21 questions. Fifteen questions focus on the needed and implemented procedures for crisis management and community resilience within the responders’ organizations. Five questions address technical challenges related to crisis management and community resilience that are faced within the responders’ organizations. For each of these questions, the survey respondents may also include additional suggestions and options. The final question in the survey aimed at prioritizing the importance of each feature.

A total of 28 organizations (all S&R partners) responded to the survey that can be classified into three major categories:

1. *First/early (F/E) responder* 8 organizations; 28.57% of responses.
2. *Technology developer (Tech)* 11 organizations; 39.29% of responses.
3. *Others* 9 organizations including communication and exploitation experts, research universities, legal and ethics expert, and so on; 32.14% of responses.

#### Gap Analysis of Findings

**Fig. 3:**
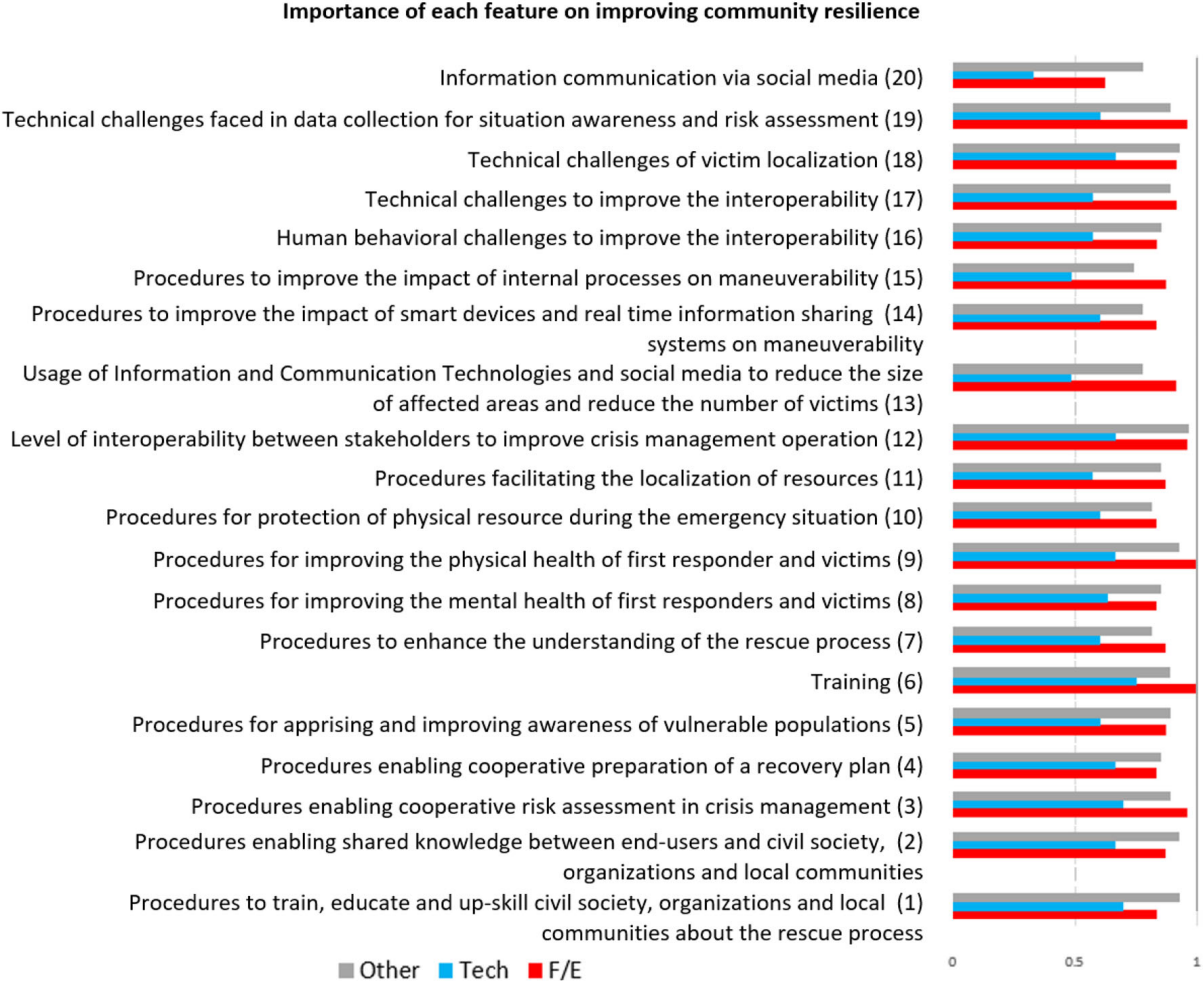
Normalized and aggregated (by group) total scores for each feature. *F/E* first/early responder, *Tech* technology developer

We segregated the collected feedback by category of respondents in order to identify different opinions among these groups. Then, we focused on the importance attributed to each feature as conveyed by the final survey question (prioritization). In this question, respondents assigned a score describing the importance of each feature (3: essential, 2: secondary, 1: auxiliary). Figure 3 shows the aggregated obtained scores by category of respondents for each feature after these have been normalized.

Next, the most important features were identified, and a list of the top-5 ranked features was created for each category. Table 1 presents the differences and similarities across the three categories for the selected features using color matching. The respective features/questions are reported in Fig. 3.

**Table 1:**
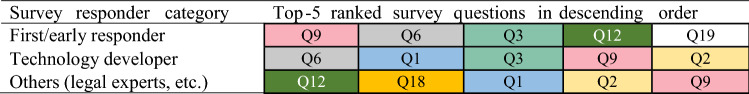
Lists of top-5 important features per category of respondents

It is interesting to note that several features, as identified by the respective color coding that are considered among the top-5 most important by the different categories, are the same for two or even all (that is, Q9) of these categories, although often positioned in different priority order. This is helpful both for understanding concerns shared across categories and for realizing subtleties and differences in the different perspective and relevant experience that pertains to each category.

**Fig. 4:**
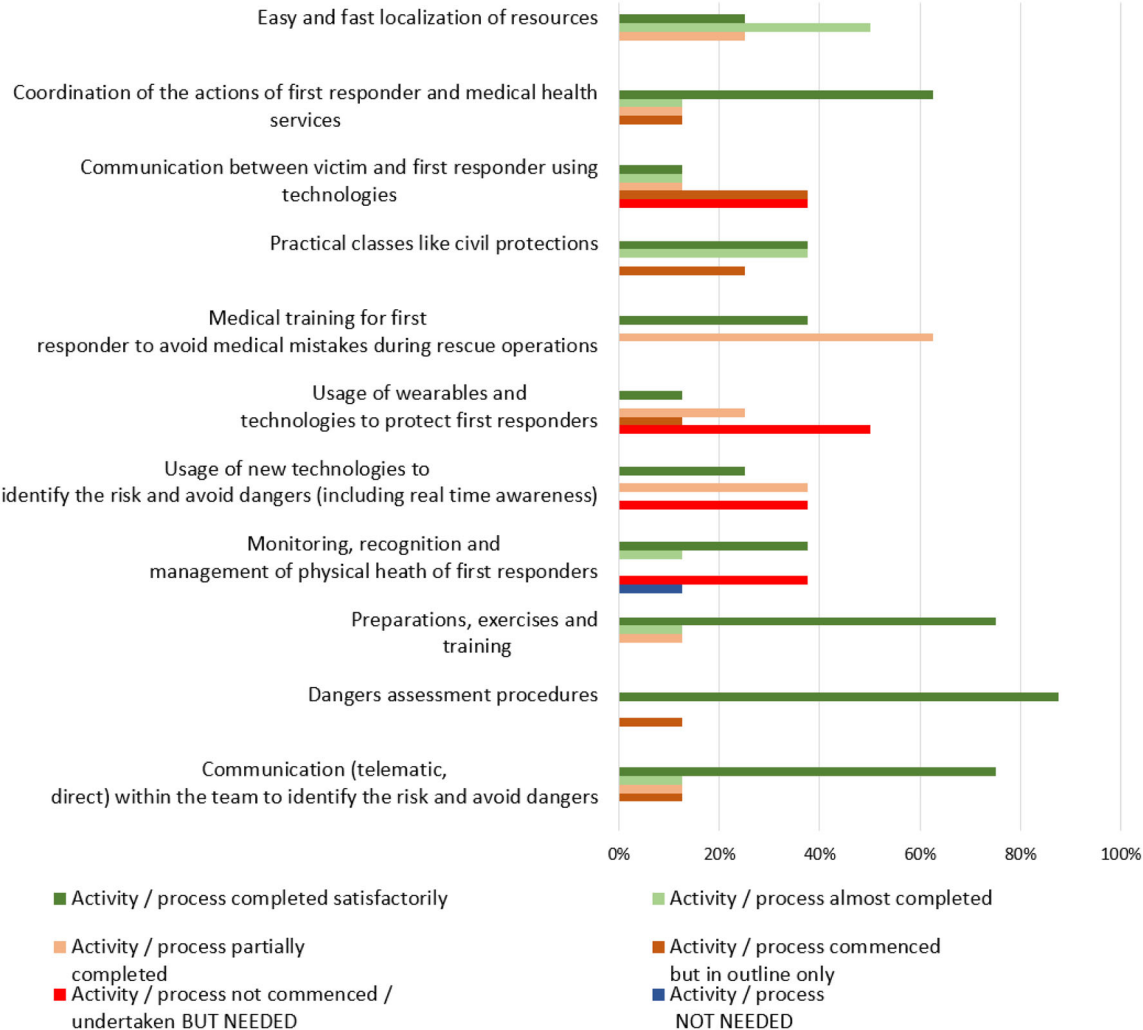
F/E responder feedback on Q9—Procedures for improving the physical health of first responder and victims

The most important features were further analyzed by identifying the related suggestions. The analysis mainly focused on F/E responders and technology developers as these possessed the most relevant experience. However, relevant feedback from others was also considered. As an example, Fig. 4 shows the F/E responders’ feedback obtained for their top-ranked feature (Q9).

The analysis revealed the importance of exploiting wearable technologies for protecting the physical health of the F/E responders. Of the F/E responder organizations, 50% described the need to use the new technologies to identify risk and avoid danger (including real time awareness) as an activity not undertaken but needed within their organizations.

The ranked list of the top-5 important features of the F/E responders is of most interest because they are the affected “community” of interest. Table 2 shows the most relevant suggestions related to the top-5 ranked features for F/E responders’ organizations.

**Table 2:**
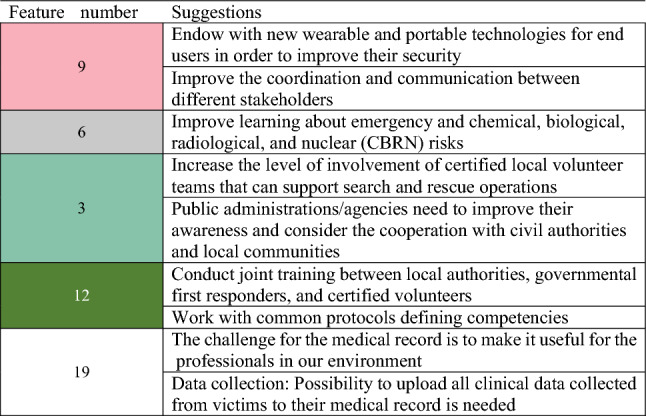
Suggestions related to the top-5 important features for first/early responders’ organizations

Next, we compared the ranked lists and relevant suggestions for the three categories of respondents in an effort to understand the reported similarities and differences. For example, the results reflected the importance attributed to training (Q6) and cooperative risk assessment (Q9) in improving community resilience. These questions were included among the top-5 priorities by both F/E responders and technology developers (Table 1). Of the F/E responder organizations, 10% stated that special training regarding interaction with people with specific disabilities needs to be included in their training programs; 20% mentioned that meetings or gamification need to be better exploited to cooperatively identify and assess risks related to crisis management.

An impression, further affirmed in the workshop that followed, was that these differences emanated from the responder’s category—different perspective and expertise on the matter—that, unavoidably, sometimes resulted in different suggestions. Capturing such different perspectives and suggestions was an added value of the approach undertaken. Limiting the survey to the 28 organizations that composed the S&R consortium offered advantages and drawbacks. Main advantages were: (1) A representative view was obtained as a composite of the 28 organizations that were well aware of the planned pilots and the relevant technologies to provide more specific and targeted feedback; and (2) The collective viewpoint limited the time needed for processing the feedback within the deadlines of the project. The main drawback was that it probably lacked the more unbiased (but probably more generic) view that could have been obtained by including organizations not related to the project. Thus, refining and validating the obtained feedback in a workshop with the participation of several external experts was deemed necessary.

#### Refinement and Validation: The Workshop Roundtable

The workshop involved participation by 305 experts in disaster management, both external and internal to the S&R consortium. It provided an opportunity to discuss the survey findings, the proposed methodology, and additional suggestions that could enhance community resilience with presentations and a dedicated roundtable discussion with key experts. The proposed approach and the presented GAP survey analysis received very positive feedback and served as a means of their validation, as well as an opportunity for further discussion and refinement of some suggestions. It also provided useful advice and revealed the positive aspects and potential limitations of the proposed approach.

In relation to key observations, some external experts have underscored the importance of sharing past experiences related to the disaster management among the involved first response entities. The impact of the Covid-19 crisis on the interoperability between national and international organizations also has been stressed. Further investigation is needed to better understand how such Covid-19 impact could be best managed.

In relation to the strengths and limitations of the proposed approach, the following list summarizes the key points:

- *Strengths* Top-down and bottom-up stakeholder-oriented participatory approach; breaks silo-thinking, facilitates communication between parties and establishes common understanding of essential concepts; easily adaptable under different context and audience; allows integration of quantitative methods (for example, multi-criteria analysis for key end user’s criteria and resilience attributes; risk analysis); and promotes creative thinking.
- *Limitations* “Expensive” in relation to expert time-commitment; requires experienced moderators in steering FG, meeting, and workshop discussions and consultations; does not provide a quantitative estimate for resilience or community resilience, although to do so was not its aim.

## Conclusion and Further Research

A participatory top-down and bottom-up approach is presented in the context of disaster management. The approach provides high-level guidance for directing the development of technologies and the operationalization of pilot testing. The article focused on the methodological approach and less on the obtained results that will be the subject of a separate work. Nevertheless, important aspects, in particular from the literature review/case study collection, the FGs and, to a lesser extent, the GAP survey, are presented. The reader is referred to the respective deliverables of the S&R project (S&R 2020a, b, 2021), for a detailed analysis of these components and of the larger project.

Due to the massive landscape of resilience, community, and community resilience definitions, concepts, perceptions, and approaches that exist in the scientific and technical literature and are encountered among the works of different practitioners and stakeholders, the proposed approach is based on concrete participatory methods that intend to reconcile and synthesize disparate views into a common understanding and vision shared among the different parties for whom improving communication and cooperation in building community resilience is a paramount objective.

In this respect, our approach is easily adaptable to different contexts, depending on the scope of work and the community considered. The participatory methods used are not exclusive and may be substituted or complemented by different ones (for example, with respect to stakeholder analysis: Strength, Weakness, Opportunity, and Threat (SWOT) analysis, Nominal Group Technique, or the Delphi technique, to name a few) depending on the process that is most commendable for establishing agreement and proceeding with decision making. Perhaps, one of our approach’s most interesting features is the interlinkage established between community resilience attributes and the key end user or community needs.

Often, resilience management frameworks and models are based on specific indicators for measuring the resilience of the studied system. The CoBRA framework (UNDP 2014), which forces consideration of the social dimension, also suggests that composite and contextually specific indicators of resilience enable us to understand how key local drivers of resilience are changing and affecting overall or universal resilience levels. Similarly, the CART (Community Resilience Advancing Toolkit) approach suggests the need for additional research on resilience metrics in order to operationalize the various resilience models. State-of-the-art research projects that deal with the resilience of critical infrastructure systems (for example, RESOLUTE; IMPROVER that proposed the Critical Infrastructure Resilience Indicator—CIRI approach; and EU-CIRCLE) emphasize the preservation or adaptation of key system functions, including societal.

Because obtaining estimates of resilience or community resilience is out of the scope of the this article, a possible direction for future research would be to consider a systematic and quantitative way to establish or integrate resilience or community resilience indicators into our proposed methodological approach or vice-versa. This would allow for the establishment of a common ground for communication and community resilience building by focusing on the appropriate indicators most relevant to the specific context.

## Footnotes

1 www.search-and-rescue.eu.

2 FP7 refers to the European Union’s (EU) 7th Framework Programme (FP) for funding research that was implemented from 2007 to 2013.

3 DRiving InnoVation in crisis management for European Resilience (DRIVER+). https://www.driver-project.eu/.

4 https://www.habitat.org/. Accessed 21 Sept 2020.

5 Global Network of Civil Society Organizations for Disaster Reduction (GDNR). https://www.gndr.org. Accessed 18 Sept 2020.

6 Search and Rescue Assistance in Disasters (SARAID). https://www.saraid.org/. Accessed Sept 18 2020 (last updated in 2017).

7 The EU project Building Resilience Across Communities in Europe (emBRACE). https://sites.google.com/site/embracefp7/home. The project ended on 30 September 2015.

8 Improved risk evaluation and implementation of resilience concepts to critical infrastructure (IMPROVER). https://cordis.europa.eu/project/id/653390.

9 Realising European ReSiliencE for CritIcaL INfraStructure (RESILENS). http://resilens.eu/.

10 Smart Mature Resilience (SMR) for more resilient cities in Europe (SMS). https://smr-project.eu/home/.

11 RESilience management guidelines and Operationalization appLied to Urban Transport Environment (RESOLUTE). http://www.resolute-eu.org/index.php.

12 DARWIN Adapt to survive—Expecting the unexpected and know how to respond. https://h2020darwin.eu/.

13 Improving Resilience to Emergencies through Advanced Cyber Technologies (i-REACT). https://cordis.europa.eu/project/id/700256.

14 A pan-European framework for strengthening Critical Infrastructure Resilience to Climate change (EU-CIRCLE). https://www.eu-circle.eu/.

15 112 is the number to call for emergencies from anywhere within the European Union.

16 https://ec.europa.eu/eusurvey/runner/2fbdde33-dde4-fa21-ae32-04d68565494c.
